# Antibodies to Periodontal Bacteria Are Associated with Systemic Lupus Erythematosus and Autoantibody Positivity

**DOI:** 10.3390/ijms262110719

**Published:** 2025-11-04

**Authors:** Laura Massarenti, Henrik Christian Bidstrup Leffers, Thorsten Brodersen, Peter Riis Hansen, Christian Damgaard, Ole Birger Pedersen, Søren Jacobsen, Claus Henrik Nielsen

**Affiliations:** 1 Institute for Inflammation Research, Center for Rheumatology and Spine Diseases, Section 7521, Copenhagen University Hospital Rigshospitalet, 2100 Copenhagen, Denmark; 2 Research Area Periodontology, Section for Oral Biology and Immunopathology, Department of Odontology, Faculty of Health and Medical Sciences, University of Copenhagen, 2200 Copenhagen, Denmark; 3 Copenhagen Center for Autoimmune Connective Tissue Diseases (COPEACT), Center for Rheumatology and Spine Diseases, Rigshospitalet, 2100 Copenhagen, Denmark; 4 Department of Clinical Immunology, Zealand University Hospital, 4600 Køge, Denmark; 5 Department of Cardiology, Herlev-Gentofte Hospital, 2900 Hellerup, Denmark; 6 Department of Clinical Medicine, Faculty of Health and Medical Sciences, University of Copenhagen, 2200 Copenhagen, Denmark

**Keywords:** systemic lupus erythematosus, *Porphyromonas gingivalis*, *Aggregatibacter actinomycetemcomitans*, smoking, periodontitis, autoantibodies

## Abstract

Periodontitis has been suggested to play a role in the etiology of systemic lupus erythematosus (SLE). However, evidence remains limited, and the underlying mechanisms are unclear. *Porphyromonas gingivalis* and *Aggregatibacter actinomycetemcomitans* are strongly linked to periodontitis. Here, we evaluate the levels of circulating antibodies against these bacteria in patients with SLE and healthy controls and analyze their association with SLE-related autoantibodies. Serum IgG antibodies against *P. gingivalis*, *A. actinomycetemcomitans* leukotoxin A (LtxA), and two control bacteria (*Capnocytophaga ochracea* and *Escherichia coli*) were quantified in 223 patients with SLE and 301 healthy controls. Data was analyzed with ANCOVA and logistic regressions adjusting for age, sex, and smoking status. Exposure to *P. gingivalis* and *A. actinomycetemcomitans*, as estimated from antibody levels, was associated with SLE (Odds Ratios (ORs) = 3.0, *p* = 0.0002 and OR = 2.61, *p* = 0.0007, respectively). An additive interaction on SLE susceptibility was observed for exposure to *P. gingivalis* and smoking, with an attributable proportion due to interaction (AP) = 0.72 (95% CI = 0.41–1.02). Anti-*A. actinomycetemcomitans* LtxA antibodies were elevated in patients positive for anti-dsDNA antibodies (*p* = 0.02) and nominally increased in those positive for anti-cardiolipin antibodies (*p* = 0.06). Elevated levels of antibodies against *P. gingivalis* and *A. actinomycetemcomitans* in patients with SLE suggest a role for these bacteria, or periodontitis, in the immunopathogenesis of SLE. An additive interaction with smoking was observed for *P. gingivalis*, and *A. actinomycetemcomitans* exposure was associated with anti–DSNA antibody positivity, supporting a link between these bacteria and SLE.

## 1. Introduction

It is well established that the etiology and clinical course of many autoimmune diseases can be influenced by bacterial infections [1,2]. Systemic lupus erythematosus (SLE) is a chronic autoimmune disease with diverse symptoms that may lead to multiorgan involvement. In addition to a strong genetic component, microbial infections have been shown to be associated with onset, exacerbation and specific clinical manifestations of SLE [3,4,5,6]. The immunological mechanisms suggested to be involved include molecular mimicry, innocent bystander activation and epitope spreading [3,7].

Dysbiosis in the oral microbiome has been reported in patients with SLE, with enrichment of some bacterial species and depletion of others, influencing disease development and course by promoting an increased systemic proinflammatory burden [8,9]. Although the association remains controversial, epidemiological studies have linked SLE with periodontitis [10,11,12,13,14], and bacterial species associated with periodontitis have been observed more frequently in patients with SLE than in healthy controls [8,14,15]. Furthermore, oral carriage of *Porphyromonas gingivalis,* one of the most virulent periodontal bacteria [16], has been reported to be associated with active SLE [8] and presence of anti-cardiolipin antibodies in patients with active disease [17]. In addition, circulating antibodies against the bacterium have been shown to be elevated in individuals positive for anti-dsDNA, independently of whether they have been diagnosed with SLE [18,19], and in patients with SLE positive for anti-Smith/RNP antibodies [19]. Circulating antibodies against *Aggregatibacter actinomycetemcomitans*, another important virulent periodontal bacterium [20], have also been shown to associate with high disease activity in SLE and seropositivity for anti-dsDNA or anti-Smith/RNP antibodies in these patients [19]. Of note, antibodies against *P. gingivalis* and *A. actinomycetemcomitans* have been reported as valid markers for oral carriage of the respective bacteria [21,22,23,24] as well as useful indicators of periodontitis [22,23,25], and are known to persist over time [24], independently of dynamic changes in bacterial load due to host responses or periodontal treatment [26,27]. Therefore, they can provide valuable information on current and previous exposure to these bacteria and the host’s systemic immune responses to them.

*P. gingivalis* and *A. actinomycetemcomitans* have both been shown to evade the immune system via multiple mechanisms. For example, *P. gingivalis* can degrade cytokines and complement components [28,29], while *A. actinomycetemcomitans* leukotoxin A (LtxA) induces leukocyte death [30], a mechanism that may contribute to prolonged antigen exposure, breakdown of immune tolerance, and autoantibody production, which are important features in the immunopathogenesis of SLE. Moreover, positive interactions between exposure to *P. gingivalis* and smoking have been reported in rheumatoid arthritis [31], suggesting that immune responses to periodontal bacteria may play a wider role in autoimmune diseases, interacting with common environmental risk factors and amplifying systemic autoimmunity. Similar mechanisms have not been addressed in SLE.

On this background, it has been hypothesized that infection with periodontal bacteria influences immune responses involved in the development and progression of SLE. Therefore, the aim of this study was to determine whether exposure to *P. gingivalis* and *A. actinomycetemcomitans*, as indicated by the presence of antibodies against these bacteria, is associated with SLE and with the presence of autoantibodies. To determine the specificity of these immune responses and rule out a generalized increase in immunoglobulin G (IgG) production often seen in SLE, antibodies against two other Gram-negative bacteria, *Capnocytophaga ochracea* (an oral bacterium) and *Escherichia coli*, were also measured. Additionally, the study explored potential interactions between exposure and host immune responses to these periodontal bacteria and smoking in relation to the presence of SLE.

## 2. Results

The demographic and clinical characteristics of patients with SLE and healthy controls included in this study are presented in Table 1. Compared to patients with SLE, healthy controls were older and had a lower proportion of females and individuals with a history of smoking (*p* < 0.0001). Although antibody levels against *P. gingivalis*, LtxA from *A. actinomycetemcomitans*, *C. ochracea*, and *E. coli* were not significantly affected by age or sex, all subsequent analyses comparing patients and controls were adjusted for these potential confounding variables. Antibodies against *P. gingivalis* were elevated in smokers compared to never smokers (*p* = 0.0075), and therefore, this variable was also included in the analyses.

### 2.1. Antibodies Against Periodontal Bacteria in SLE and in Healthy Individuals

**Figure 1:**
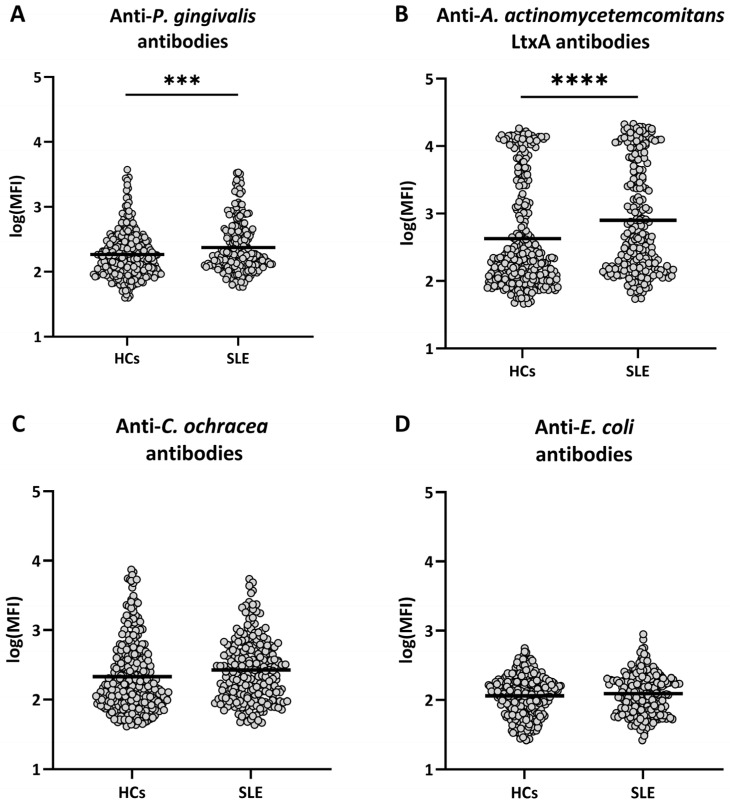
Levels of antibodies to the selected bacteria in healthy controls and patients with SLE. Serum samples from 301 healthy controls (HCs) and 223 patients with SLE were assessed for antibodies against (**A**) *P. gingivalis*, (**B**) leukotoxin A (LtxA) from *A. actinomycetemcomitans*, (**C**) *C. ochracea*, and (**D**) *E. coli* by means of Luminex technology. Log10 of the median fluorescence intensity (MFI) values are shown, with horizontal bars representing mean values. Differences were analyzed with ANCOVA with adjustments for age, sex and smoking status. *** = *p* < 0.001, **** = *p* < 0.0001.

Levels of antibodies against *P. gingivalis* were elevated in patients with SLE compared to healthy controls (*p* = 0.0002, Figure 1A). Likewise, the level of antibodies against *A. actinomycetemcomitans* LtxA was higher in patients with SLE than in healthy controls (*p* < 10^−5^, Figure 1B). On the other hand, no significant differences in levels of antibodies against *C. ochracea* and *E. coli* were observed (Figure 1C,D).

We and others have previously reported that levels of antibodies against *P. gingivalis* and *A. actinomycetemcomitans* LtxA are reliable indicators of current oral carriage of the respective bacterium [21,22,23]. Furthermore, levels of antibodies against these periodontal bacteria are known to be stable over time [24]. Cut-off levels derived from our previous studies [22,23] were applied to measurements from this study to identify oral carriers and non-carriers of *P. gingivalis* and *A. actinomycetemcomitans*.

Patients with SLE were significantly more likely to be oral carriers of *P. gingivalis* than healthy controls (OR = 3.02, *p* = 0.0002). Similarly, carriage of *A. actinomycetemcomitans* was more common among patients with SLE than among healthy controls (OR = 2.61, *p* = 0.0007).

### 2.2. Antibodies Against Periodontal Bacteria and Smoking Status in SLE Etiology

Smoking is recognized as a major risk factor for SLE [32,33], and carriage of periodontal bacteria is emerging as a possible risk factor for the disease [34]. Therefore, we examined whether exposure to *P. gingivalis* or *A. actinomycetemcomitans*, determined by increased levels of antibodies against the bacteria, interacts with smoking to influence disease susceptibility. As reported in Table 2, carriage of *P. gingivalis* and a history of smoking both increased the likelihood of having SLE (OR = 2.65, *p* < 0.01 and OR = 10.34, *p* < 10^−15^, respectively), but the combination of these risk factors increased the odds ratio dramatically (OR = 42.48, *p* < 10^−11^) with an attributable proportion (AP) due to this interaction of 0.72 (95% CI: 0.41–1.02). Likewise, the likelihood of having SLE was increased both by carriage of *A. actinomycetemcomitans* (OR = 2.91, *p* < 0.001) and smoking (OR = 13.57, *p* < 10^−19^), while the OR was even higher after exposure to both risk factors (OR = 23.29, *p* < 10^−7^). However, the apparent cumulative interaction between smoking and oral carriage of *A. actinomycetemcomitans* was not significant (AP = 0.34, 95%CI = −0.42–1.09).

### 2.3. Antibodies Against Periodontal Bacteria in Relation to SLE Autoantibodies

The levels of antibodies against *P. gingivalis* did not differ between patients with SLE, who were seronegative or seropositive for anti-dsDNA, anti-Smith, anti-U1RNP or anti-cardiolipin antibodies. The same was observed for antibodies against *E. Coli*. In contrast, patients who were seropositive for anti-dsDNA antibodies had higher levels of antibodies against *A. actinomycetemcomitans* LtxA than patients who were seronegative for anti-dsDNA autoantibodies (*p* = 0.02). A similar trend towards elevated levels of antibodies against *A. actinomycetemcomitans* LtxA was observed with respect to seropositivity for anti-cardiolipin antibodies (*p* = 0.06). Of note, the level of antibodies against *C. ochracea* also showed a tendency toward being increased in patients who were seropositive for anti-cardiolipin antibodies (*p* = 0.14).

Carriers of *A. actinomycetemcomitans*, identified based on increased antibody levels against LtxA, tended to be more likely to have anti-cardiolipin antibodies than non-carriers, although this association was not statistically significant after adjustment (OR: 1.80, *p* = 0.08).

## 3. Discussion

There is evidence to support an association between SLE and exposure to periodontal bacteria [10,11], but the underlying mechanisms remain unclear. Antibodies against *P. gingivalis* and *A. actinomycetemcomitans*, two pathogens strongly associated with periodontitis, are well-established markers of bacterial carriage and infection, as well as of active periodontal disease [21,22,23]. These antibody levels are remarkably stable over time, persisting for at least 15 years [24]. We therefore examined levels of antibodies against these two periodontal bacteria in relation to SLE diagnosis and seropositivity for SLE autoantibodies. Moreover, to our knowledge, we investigate for the first time the interaction between systemic immunoreactivity against periodontal bacteria and smoking in SLE.

We observed elevated levels of circulating antibodies against *P. gingivalis* in patients with SLE compared to healthy controls. This is in accordance with the findings of a study by Bagavant et al. [19], while no significant difference between the two groups was found in another study [18]. Similarly, we observed higher levels of circulating antibodies against LtxA from *A. actinomycetemcomitans* in patients with SLE than in healthy controls, while this was not observed in the study by Bagavant et al. [19]. The lack of concordance between the studies may be due to differences in the selected antigens used (e.g., selected virulence factors vs. whole bacteria) and the specifics of the study cohorts. However, the sum of evidence supports the notion of a link between SLE and oral infection with *P. gingivalis* or *A. actinomycetemcomitans*. Indeed, individuals identified as oral carriers of *P. gingivalis* or *A. actinomycetemcomitans* in our study, based on elevated levels of antibodies against these bacteria, were also more likely to have SLE than non-carriers. Importantly, the levels of antibodies against *C. ochracea* and *E. coli* did not differ between patients with SLE and healthy controls, suggesting that the reported association between antibodies against *P. gingivalis* or *A. actinomycetemcomitans*, on the one hand, and SLE, on the other hand, is likely due to immune responses against these two bacteria, specifically. Indeed, several independent mechanisms have been suggested to explain associations between bacterial infection and autoimmune disease, including molecular mimicry, innocent bystander activation and epitope spreading [3,7].

We found that the susceptibility to SLE was substantially elevated when oral carriage of *P. gingivalis*, as indicated by high levels of antibodies against the bacterium, coincided with smoking, compared to either of the factors alone, and an additive interaction between the two was observed. A similar pattern was observed for oral carriage of *A. actinomycetemcomitans* in combination with smoking, although this interaction was not statistically significant. These risk factors can independently promote immune dysregulation, chronic inflammation, and autoantibody production [35,36,37]. Their combined effect likely acts synergistically to enhance autoimmunity, consistent with mechanisms observed in other autoimmune diseases such as rheumatoid arthritis [31,38]. These findings suggest that systemic immunoreactivity to periodontal pathogens, particularly in combination with smoking, may contribute to the immunopathogenesis of SLE, although causality cannot be inferred from this cross-sectional study.

We did not observe differences in the levels of antibodies against *P. gingivalis* between patients who were seropositive and those who were seronegative for the examined autoantibodies. In contrast, two studies have reported increased levels of antibodies against *P. gingivalis* in patients with SLE seropositive for anti-dsDNA and anti-Smith/RNP antibodies [18,19]. On the other hand, the levels of antibodies against *A. actinomycetemcomitans* LtxA were higher in patients who were seropositive for anti-dsDNA antibodies than in patients without these antibodies, which agrees with what has been reported in a previous study [19]. Furthermore, the presence of high levels of antibodies against *A. actinomycetemcomitans* was associated with seropositivity for anti-cardiolipin antibodies. Indeed, high levels of anti-cardiolipin antibodies in patients with active SLE harboring *P. gingivalis* in the oral cavity, compared with patients with active disease without the bacteria, have been reported [17], but we did not confirm this finding. Accordingly, although some discrepancies between available studies exist, increasing evidence suggests a role for periodontal bacteria and the associated host immune responses in promoting the formation of autoantibodies against SLE-associated autoantigens such as dsDNA and cardiolipin. In particular, LtxA from *A. actinomycetemcomitans* is a virulence factor leading to the lysis of neutrophils with a consequent release of intracellular components, such as nuclear proteins and dsDNA, which are known autoantigens [38]. About 15–20% of patients with periodontitis have elevated levels of anti-cardiolipin antibodies, which is a higher percentage than in individuals without periodontal disease [39,40]. This is thought to result, in part, from molecular mimicry between antigens from periodontal bacteria, including *A. actinomycetemcomitans*, and β2-glycoprotein I [41,42].

Some limitations of our study should be considered. For example, our measure of bacterial carriage relied on antibody levels, which are established indicators of oral exposure, but may introduce some misclassification or bias. However, since circulating antibodies are a more accurate measure of systemic immune reactions to the periodontopathogenic bacteria and persist for a long period of time [24], they may be more closely related to systemic immunoreactivity to bacteria and autoimmune reactions than to ongoing bacterial infection. In addition, the lack of data on SLE disease activity scores limited our ability to assess whether bacterial exposure correlates with disease severity. Notably, the cross-sectional design precludes any conclusions about causality, and given that carriage of *P. gingivalis* and *A. actinomycetemcomitans* is often associated with periodontal disease, the observed association between carriage of periodontal pathogens and SLE might in part be due to periodontitis contributing to systemic inflammation, e.g., through the release of proinflammatory cytokines into the bloodstream. The strengths of the study include a relatively large, well-characterized SLE cohort and adjustment for key confounding variables.

In conclusion, our findings indicate that systemic immunoreactivity to *P. gingivalis* and *A. actinomycetemcomitans* is associated with SLE, and that smoking strongly amplifies the association. Moreover, antibodies to *A. actinomycetemcomitans* were linked to autoantibody (anti–dsDNA and anti-cardiolipin) positivity, suggesting a potential role for these bacteria, or the host immune response they elicit, in SLE pathogenesis. Because clinical periodontal status was not assessed, we cannot determine whether the observed associations reflect systemic immunoreactivity to the bacteria or the release of proinflammatory molecules from the site of periodontal inflammation into the circulation. Prospective studies incorporating clinical and microbiological assessments are warranted to clarify causality.

## 4. Materials and Methods

### 4.1. Patients and Healthy Controls

This study was based on serum samples from a cohort of 223 patients with SLE and 301 healthy controls. Samples from patients with SLE were collected at Copenhagen University Hospital, Rigshospitalet, as previously reported [43]. The diagnosis was made based on the classification criteria by the American College of Rheumatology [44,45]. Approval for the use of samples from patients with SLE was obtained from the Scientific Ethics Committee for the Capital Region of Denmark (KF 01-024/03 and KF01261669). Serum samples from healthy controls were collected at the Zealand Region blood donation facilities and included donors participating in the Danish Blood Donor Study [46] under approval by the Regional Committee on Health Research Ethics for Zealand (SJ-740). All donors gave their written and informed consent, and the study was conducted in compliance with the Declaration of Helsinki.

### 4.2. Luminex Multiplex Assay for Anti-Bacterial Antibodies

Heath-inactivated *P. gingivalis* and LtxA from *A. actinomycetemcomitans* were coupled to xMAP beads (Luminex Corporation, Austin, TX, USA) following the manufacturer’s instructions. In addition, two other Gram-negative bacteria, heat-inactivated *Capnocytophaga ochracea* and *Escherichia coli,* were also coupled to beads to serve as controls, and one blank bead set without antigens was included in the multiplex assay to identify patients with antibody reactivity against the beads per se. Antibody levels were evaluated in serum samples, as previously described [22,23]. In brief, diluted serum samples were incubated with antigen-coupled beads, and following incubation with biotinylated anti-human IgG (cat: AB3773, Sigma-Aldrich, St. Louis, MO, USA) and streptavidin–phycoerythrin (cat: PJ31S, ProZyme, Hayward, CA, USA), fluorescence was measured using a Bio-Plex 200 system (Bio-Rad, Hercules, CA, USA). A panel of samples showing different levels of anti-*P. gingivalis* and anti-*A. actinomycetemcomitans* LtxA antibodies was included in each plate run as inter-plate calibrators (inter-plate coefficient of variation ≤ 15%).

### 4.3. Autoantibody Measurement

SLE-associated autoantibodies had been measured prior to the present study in samples from the patients with SLE as part of the routine diagnostic and follow-up. These measurements were performed using established immunoassay techniques, most commonly enzyme-linked immunosorbent assays (ELISAs). These assays are standard, validated methods widely applied in both clinical and research settings, ensuring high reproducibility and comparability with other studies.

### 4.4. Statistical Analysis

All calculations were made in RStudio Version 2023.06.2 Build 561 (RStudio Inc., Boston, MA, USA) using R version 4.2.1 (R Foundation for Statistical Computing, Vienna, Austria), and graphical representation of the data was performed with GraphPad Prism 10 software (GraphPad Software, San Diego, CA, USA). All analyses were performed on log10-transformed values of antibody levels against the four bacteria. Differences in antibody levels between patients with SLE and healthy controls, as well as between patients seronegative or seropositive for SLE-associated autoantibodies, were evaluated with ANCOVA, with age, sex and smoking status (indicated as a history of previous or current smoking) as covariates. Cut-off levels indicating individuals who were likely harboring *P. gingivalis* or *A. actinomycetemcomitans* in the oral cavity based on elevated antibody levels against the two bacteria were obtained from previous studies from our group, evaluating serum antibody levels and presence of the corresponding bacteria in saliva samples, via qPCR [22,23]. Associations between oral carriage of the bacteria and the SLE diagnosis or autoantibody positivity were determined using logistic regression to estimate ORs, with adjustments for age, sex and smoking status. The additive interaction between carriage of periodontal bacteria and ever-smoking status in SLE etiology was evaluated with the “epiR” package in R, with a calculation of the attributable proportion due to interaction (AP), which is equal to 0 if there is no biological interaction [47]. The level of significance was set at *p* < 0.05.

## Figures and Tables

**Table 1:** Demographic characteristics of patients with systemic lupus erythematosus (SLE) and healthy controls.

	SLE (n = 223)	Healthy Controls (n = 301)	*p*-Value
Age, median years (range)	40 (17–72)	48 (20–71)	**<10^−4^**
Women, n (%)	201 (90%)	198 (66%)	**<10^−4^**
Ever smokers, n (%)	122 (55%)	42 (14%)	**<10^−4^**
Anti-dsDNA antibodies +, n (%)	176 (79%)	n.a.	
Anti-Smith antibodies +, n (%)	26 (12%)	n.a.	
Anti-U1RNP antibodies +, n (%)	36 (16%)	n.a.	
Anti-cardiolipin antibodies +, n (%)	109 (49%)	n.a.	

dsDNA: double-stranded DNA, U1RNP: U1 ribonucleoprotein. n.a.: not available. Difference in age between patients with SLE and healthy controls was evaluated with Mann–Whitney test, while differences between groups for binary variables were compared with χ2-test. Significant differences are shown in bold.

**Table 2:** Interaction between smoking and exposure to periodontal bacteria (on the basis of levels of antibodies against the bacteria) in the etiology of systemic lupus erythematosus (SLE).

Risk Factors		OR	95% CI	*p*-Value
Oral Carriage	Ever Smoker			
***P. gingivalis***				
-	-	1 (ref)		
+	-	**2.65**	**1.33–5.29**	**<0.01**
-	+	**10.34**	**5.91–18.08**	**<10^−15^**
+	+	**42.48**	**14.5–124.58**	**<10^−11^**
AP	**0.72 (0.41–1.02)**			
***A. actinomycetemcomitans***				
-	-	1 (ref)		
+	-	**2.91**	**1.57–5.40**	**<0.001**
-	+	**13.57**	**7.74–23.77**	**<10^−19^**
+	+	**23.29**	**7.43–72.97**	**<10^−7^**
AP	0.34 (−0.42–1.09)			

OR: odds ratio, CI: confidence interval, AP: attributable proportion due to interaction. ORs were calculated with logistic regression adjusted for age and sex, and AP was calculated with the “epiR” package in R. Significant differences are shown in bold.

## Data Availability

The datasets used and/or analyzed during the current study are available from the corresponding author on reasonable request.

## Abbreviations

The following abbreviations are used in this manuscript:

AP	Attributable proportion due to interaction
dsDNA	Double-stranded DNA
IgG	Immunoglobulin G
LtxA	Leukotoxin A
OR	Odds ratio
SLE	Systemic lupus erythematosus
U1RNP	U1 ribonucleoprotein
